# Efficacy and safety of HSK21542 for pruritus management in hemodialysis patients: a multicenter, randomized, double-blind, placebo-controlled trial

**DOI:** 10.3389/fphar.2025.1583515

**Published:** 2025-06-24

**Authors:** Ming-Ming Pan, Min Gao, Li Zhou, Yan Xu, Li Yao, Chao-Qing Wu, Chang-Lin Mei, Zhan-Zheng Zhao, Dong Sun, Tian-Jun Guan, Qin-Kai Chen, Ming Shi, Hui Xu, Ya-Ming Li, Wan-Yun Zhao, Rui Yan, Bi-Cheng Liu

**Affiliations:** ^1^ Institute of Nephrology, Zhong Da Hospital, Southeast University School of Medicine, Nanjing, Jiangsu, China; ^2^ Department of Nephrology, Kidney Research Institute, West China Hospital of Sichuan University, Chengdu, Sichuan, China; ^3^ Department of Nephrology, The Affiliated Hospital of Qingdao University, Qingdao, Shandong, China; ^4^ Department of Nephrology, The First Hospital of China Medical University, Shenyang, Liaoning, China; ^5^ Department of Nephrology, The People’s Hospital of Guangxi Zhuang Autonomous Region, Nanning, Guangxi, China; ^6^ Department of Nephrology, Changzheng Hospital, Second Military Medical University, Shanghai, China; ^7^ Department of Nephrology, Zhabei Central Hospital of JingAn District of Shanghai, Shanghai, China; ^8^ Department of Nephrology, The First Affiliated Hospital of Zhengzhou University, Zhengzhou, Henan, China; ^9^ Department of Nephrology, Affiliated Hospital of Xuzhou Medical University, Xuzhou, Jiangsu, China; ^10^ Department of Nephrology, Zhongshan Hospital, Xiamen University, Xiamen, Fujian, China; ^11^ Department of Nephrology, The First Affiliated Hospital of Nanchang University, Nanchang, Jiangxi, China; ^12^ Department of Nephrology, Renmin Hospital of Wuhan University, Wuhan, Hubei, China; ^13^ Department of Nephrology, Xiangya Hospital of the Central South University, Changsha, Hunan, China; ^14^ Haisco Pharmaceutical Group Co., Ltd., Chengdu, Sichuan, China; ^15^ Department of Nephrology, Affiliated Hospital of Guizhou Medical University, Guiyang, Guizhou, China

**Keywords:** CKD-aP, HSK21542, hemodialysis, κ-opioid receptor agonist, uremic pruritus

## Abstract

**Background:**

Chronic kidney disease-associated pruritus (CKD-aP) is a common and distressing symptom in hemodialysis patients. This Phase II trial evaluated the efficacy and safety of HSK21542, a selective kappa-opioid receptor agonist, in managing CKD-aP.

**Methods:**

Adult patients on hemodialysis with moderate to severe pruritus, were randomized 1:1:1 to placebo, or HSK21542 (0.3 μg/kg or 0.6 μg/kg) administered thrice weekly post-dialysis for 12 weeks. The primary endpoint was the change from baseline in the weekly mean of the worst itching intensity Numerical Rating Scale (WI-NRS) score at week 12. Secondary endpoints included quality-of-life assessments, safety evaluations, and pharmacokinetic properties.

**Results:**

A total of 90 patients were enrolled. At week 12, mean changes in WI-NRS scores from baseline were −2.94 for the placebo group, −3.40 for the 0.3 μg/kg HSK21542 group, and −2.21 for the 0.6 μg/kg HSK21542 group. The percentages of patients who had a reduction of 3 points or above in their WI - NRS scores were 44.4% in the placebo group, 62.1% in the 0.3 μg/kg HSK21542 group, and 37.0% in the 0.6 μg/kg HSK21542 group. The 0.30 μg/kg HSK21542 group demonstrated more significant improvements in Skindex - 16 scores compared to the placebo. The 5-D Itch Scale scores also presented similar trends. Both the 0.3 μg/kg and 0.6 μg/kg doses of HSK21542 were well - tolerated, with no dose-dependent adverse effects.

**Conclusion:**

The 0.3 μg/kg dose of HSK21542 demonstrated superior efficacy and safety in reducing pruritus and improving quality of life in hemodialysis patients.

## 1 Introduction

Chronic kidney disease - related pruritus (CKD - aP), or uremic pruritus, commonly affects patients with end - stage renal disease (ESRD) on hemodialysis. Its prevalence varies widely, from 20% to 80%. This condition is common and causes significant discomfort among this patient group (Agarwal et al., 2021a; Metzger et al., 2021). CKD - aP can cause sleep problems, depression, and raise the risk of cardiovascular diseases (Nair and Finkelstein, 2020; Makar et al., 2021). Despite various treatment options, including topical therapies and systemic medications, no standardized guidance exists for its management, making effective treatment of CKD-aP challenging (Simonsen et al., 2017; Martin et al., 2020).

Mechanisms involve the build-up of uremic toxins, peripheral neuropathy, immune system malfunction, and opioid imbalance (Verduzco and Shirazian, 2020). Recent research indicates that excessive activation of central mu-opioid receptors (MOR) or inhibition of peripheral kappa-opioid receptors (KOR) could play a role in triggering the itching sensation (Jha et al., 2022; Kim et al., 2022). Notably, a significant decrease in KOR expression has been observed in the skin of CKD-aP patients (Wieczorek et al., 2020), indicating that KOR agonists may have therapeutic potential for CKD-aP treatment. KOR agonists such as nalfurafine (Wikström et al., 2005; Kumagai et al., 2010; Zhang et al., 2023) have been shown to be effective in reducing pruritus but are often associated with significant central nervous system side effects. Difelikefalin is a selective KOR agonist with substantial efficacy in the management of CKD-associated pruritus. Clinical trials revealed its capability to markedly reduce pruritus intensity, enhance sleep quality, and elevate overall patient quality of life. Nonetheless, the treatment group experienced a notably higher incidence of gastrointestinal adverse events (AEs) compared to the placebo (Fishbane et al., 2020b; Yosipovitch et al., 2023). These observations provide valuable insights for the development of new agents.

HSK21542, a novel selective KOR agonist, has shown promising results in preclinical and early-phase clinical studies, with a favorable safety profile and potential for long-term use. HSK21542 represents a new kind of synthetic short - chain polypeptide. It has the property of selectively acting as an agonist for peripheral kappa - opioid receptors (KOR). Its unique molecular structure provides high selectivity and affinity (Wang et al., 2021), offering potential therapeutic advantages by modulating potassium and calcium ion currents through the involvement of G proteins. HSK21542 efficiently halts the transmission of pain and itch signals. It curbs the excitability of dorsal root ganglia and peripheral sensory nerves while decreasing the release of inflammatory factors and neurotransmitters. These mechanisms contribute to its analgesic and itch-inhibiting effects without activating receptors outside KOR, indicating better safety and a higher level of tolerability in contrast to other opioid agonists (Violin et al., 2014; Wang et al., 2021; Pan et al., 2023).

We conducted a phase II trial with a two-stage design. The first stage involved a dose-escalation study to assess the safety and how well HSK21542 was tolerated during a 1-week treatment span. This study established that HSK21542 was well-tolerated within the 0.05–0.80 μg/kg dose range, with multiple intravenous administrations of increasing doses to identify the optimal dose (Pan et al., 2023). Here, we report the 12-week Phase II study evaluating the clinical efficacy, safety, and pharmacokinetics of HSK21542.

## 2 Methods

### 2.1 Study design and patients

This study expanded upon the preliminary findings of the first stage of our Phase II trial (Pan et al., 2023) to assess the efficacy and safety of HSK21542 injection over a 12-week treatment period in alleviating moderate to severe pruritus in hemodialysis patients. This trial was designed identify the optimal dosage and administration regimen for potential advancement into a Phase III clinical trial.

Eligible participants were adults with ESRD undergoing hemodialysis thrice weekly for at least 3 months with a dry weight between 45.0 and 135.0 kg and patients needed to have a body mass index falling within the range of 16.0–30.0 kg/m^2^. Those who had moderate to severe pruritus, which was determined as having a weekly mean of the Worst Itching Intensity Numeric Rating Scale (WI - NRS) score exceeding four in daily evaluations carried out in the 7 - day run - in period prior to randomization, were eligible for inclusion. To ensure adequate dialysis, patients were also required to have, within 6 months prior to dosing, and they should have either at least two measurements of the single - pool urea clearance index (sp Kt/V) being greater than or equal to 1.2, or at least two urea reduction rates (URR) of 65% or higher. An alternative qualifying scenario is having one measurement of sp Kt/V ≥ 1.2 and one measurement of URR ≥65%. Urea clearance parameters (sp Kt/V and URR) are important indicators of hemodialysis efficiency and reflect the patient’s renal function status and toxin clearance capacity. CKD-aP is associated with multiple factors, including toxin accumulation, peripheral neuropathy, and immune system dysfunction. Inadequate toxin clearance can exacerbate pruritus symptoms. (Verduzco and Shirazian, 2020). Therefore, ensuring adequate dialysis through these parameters is crucial for evaluating the efficacy of HSK21542 in managing pruritus. Exclusion criteria included patients scheduled for kidney transplantation or parathyroidectomy and those whose itching was unrelated to ESRD or its complications, such as pruritic skin diseases or cholestatic liver diseases. In addition, patients who had started or changed medications known to affect itch, For example, medications like antipsychotics, sedative - hypnotics, and selective serotonin reuptake inhibitors (SSRIs), anti-anxiety drugs, or tricyclic antidepressants, within 14 days prior to screening were excluded.

The research was conducted in accordance with the ethical tenets of the Declaration of Helsinki and Good Clinical Practice (GCP). The ethics committees of all participating research centers approved the study, as detailed in the previously published manuscript (Pan et al., 2023). Before initiating any study - related procedures, written informed consent was secured from every participant. The trial is registered with ClinicalTrials.gov (identifier NCT04470154).

### 2.2 Study procedure, randomization, and blinding

The study consisted of a 1 - week introduction phase, a 12 - week treatment phase, and a 2 - week follow - up phase. Patients were randomly assigned in a 1:1:1 proportion to receive either a placebo, HSK21542 at a 0.3 μg/kg dosage, or HSK21542 at a 0.6 μg/kg dosage. Randomization was conducted using a computer-generated sequence by an independent statistician to ensure allocation concealment. The study was double-blinded, meaning that neither the patients nor the investigators knew the treatment assignments.

**FIGURE 1:**
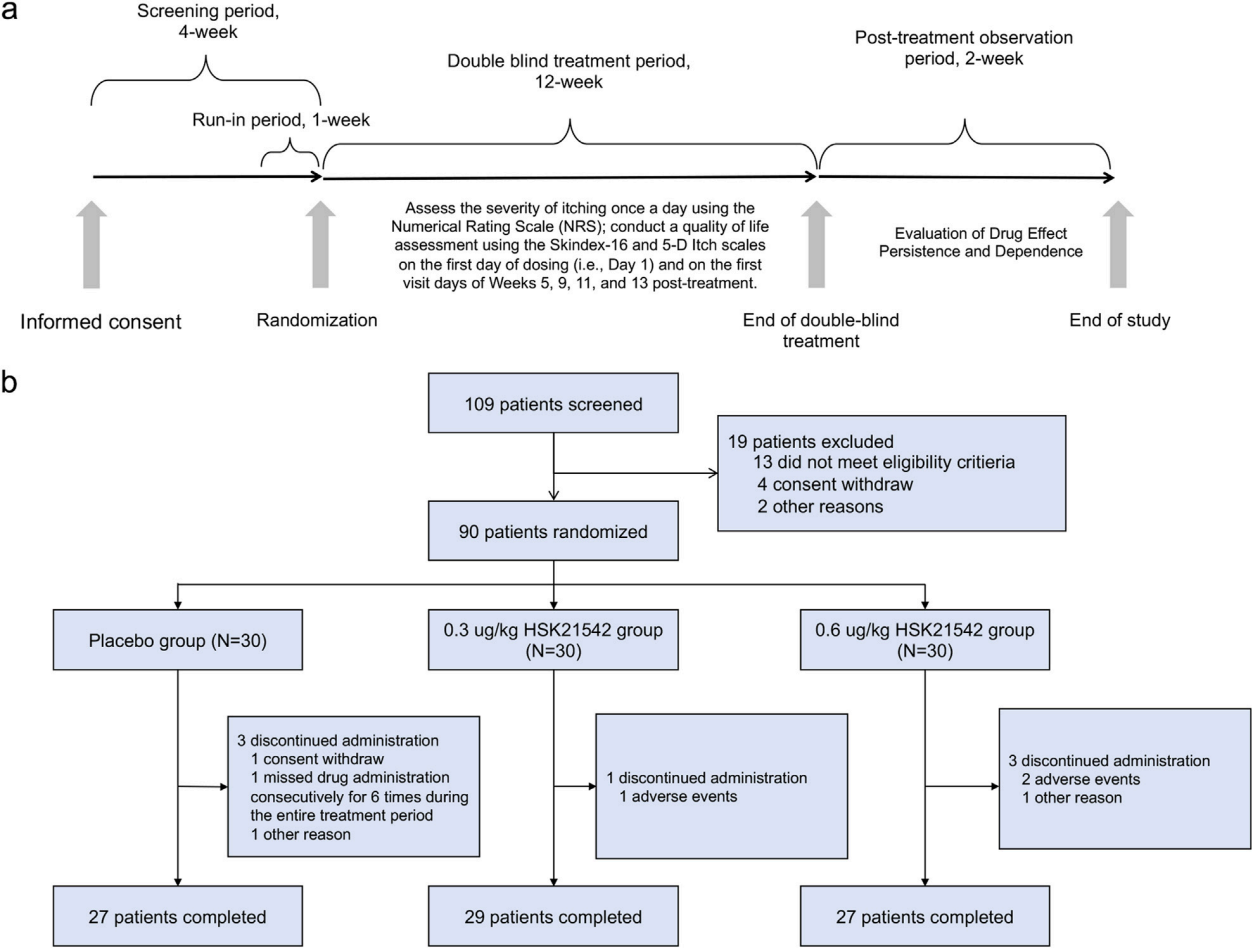
**(a)** Overall study design. **(b)** Enrollment, Randomization, and Treatment Assignment.

Following randomization, patients received their assigned treatment after each dialysis session on Days 1, 3 (+2), and 5 (+2) for 12 consecutive weeks. The study drug was given intravenously within 10 min after the dialysis session ended, based on the patient’s dry weight and assigned dose group (Figure 1a).

### 2.3 Endpoints assessments

The primary endpoint was the change in the weekly mean of the daily WI - NRS score at week 12, measured relative to the baseline. Secondary endpoints were formulated to delve deeper into how the treatment affected itch intensity and quality of life (QoL). In particular, we examined the percentage of patients who experienced a reduction of at least 3 points and at least four points from the baseline WI - NRS score at week 12. Additionally, we analyzed the changes in QoL as measured by the Skindex - 16 and 5 - D Itch scales at week 12. Safety evaluations involved tracking the occurrence of AEs and serious adverse events (SAEs). Furthermore, additional assessments included the evaluation of itch severity after discontinuation of the study drug, measured by the change in the weekly mean the WI-NRS scores, and the risk of physical dependence were assessed using the Short Opiate Withdrawal Scale (ShOWS) and Objective Opioid Withdrawal Scale (OOWS) scores during the first 2-week post-treatment period. The dose-response relationship was analyzed to explore correlations between the doses of HSK21542, average maximum concentrations (C_max_), and fluctuations in the weekly mean score of the WI-NRS as compared to the baseline level.

### 2.4 Statistical analysis

The sample size for the second stage of our Phase II trial was determined based on practical considerations and insights from preliminary data obtained in Phase I (Wang et al., 2021) and the first stage of Phase II studies (Pan et al., 2023). After thorough deliberation by the study team, it was decided that the second stage would enroll 90 patients, with 30 patients equally allocated to each of the three study groups: placebo, HSK21542 at a dose of 0.3 μg/kg, and 0.6 μg/kg. This sample size was selected to provide a robust dataset for assessing the efficacy and safety of HSK21542 while remaining feasible within the constraints of the study resources and timeline. The analysis primarily comprised the Full Analysis Set (FAS), this included all randomized patients who had received at least one dose of the study medication and had completed at least one efficacy assessment after baseline. The discontinuation observation analysis set was used to analyze the ShOWS and OOWS scores within the 2-week discontinuation period. Descriptive statistics summarized WI-NRS for itching intensity at baseline and subsequent visits. The primary endpoint was analyzed through the application of a Mixed-Effects Model for Repeated Measures (MMRM). This model incorporated fixed effects for the treatment group, visit, and their interaction, with participants as a random effect and baseline WI-NRS as a covariate. The least squares (LS) mean changes from the baseline and their corresponding 95% confidence intervals were computed for each treatment group. Sensitivity analyses were performed to assess the robustness of the primary endpoint under diverse missing-data assumptions and imputation algorithms. To handle missing data, we performed multiple imputations on the missing values of the weekly average NRS at each analysis visit during the clinical trial using proc mi in SAS. For the assessment of QoL scores, including the Skindex-16 and 5-D Itch scales, an MMRM was applied, consistent with the primary endpoint analysis. Between-group comparisons of the LS mean changes were also examined. All analyses were conducted using SAS software, version 9.4, to ensure compliance with the study’s statistical methods.

## 3 Results

In total, 109 patients were screened, with 90 patients randomly assigned to the treatment groups. placebo, 0.30 μg/kg HSK21542, and 0.60 μg/kg HSK21542 groups, with 30 participants in each group. All 90 enrolled patients received the study medication and were admitted to the FAS and Safety Set (SS). Eighty-three patients were included in the discontinuation observation set (Figure 1b). Most participants were male (70–80%). Dry weights varied minimally across the groups, with mean values ranging from approximately 65–67 kg. The baseline weekly WI-NRS score, indicating moderate to severe itch, consistently ranged from 6.3 to 6.5. The Skindex-16 scores ranged from approximately 41.6–51.6, while the 5-D Itch scores fluctuated between 15.1 and 16.1 (Table 1). Populational and baseline characteristics were consistent across all treatment groups.

**TABLE 1:** Baseline demographics and disease characteristics*.

	Placebo (N = 30)	HSK21542 0.3 μg/kg (N = 30)	HSK21542 0.6 μg/kg (N = 30)
Sex, No. (%)			
Male	24 (80.0)	21 (70.0)	23 (76.7)
Female	6 (20.0)	9 (30.0)	7 (23.3)
Age, mean (SD), y	56.5 (11.7)	55.2 (11.6)	53.0 (11.3)
Height, mean (SD), cm	168.3 (7.8)	167.2 (7.5)	166.7 (8.0)
Dry weight, mean (SD), kg §	66.7 (10.8)	65.7 (9.6)	63.1 (10.7)
BMI, mean (SD)	23.4 (2.7)	23.4 (2.7)	22.6 (3.0)
Single-pool urea clearance index (sp Kt/V) ≥1.2	1.2 (0.9)	1.1 (1.0)	1.3 (1.0)
Urea reduction rate (URR) ≥65%	1.2 (1.0)	1.6 (0.9)	1.6 (0.8)
sp Kt/V ≥1.2 and URR ≥65%	1.0 (1.0)	1.0 (0.93)	1.1 (0.9)
Weekly WI-NRS score ††, mean (SD)	6.5 (1.7)	6.5 (1.6)	6.3 (1.5)
Skindex-16 scale score §§, mean (SD)	41.6 (23.3)	44.4 (26.5)	51.6 (19.2)
5-D Itch scale score ‡‡, mean (SD)	16.1 (3.8)	15.1 (3.8)	15.7 (3.7)

*SD: standard deviation

§The prescription dry body weight for dialysis is the weight at which the patient’s volume status is neither overhydrated nor underhydrated, according to the physician’s determination; at each dialysis session, fluid is removed to try to lower the patient’s weight to this prescribed dry weight.

†† Worst itching intensity was evaluated by means of the Worst Itching Intensity Numerical Rating Scale (WI-NRS), an 11-point scale that measures the worst itching intensity during the previous 24 h; scores range from 0 to 10, with higher scores indicating greater intensity.

§§ Scores on the Skindex-16, scale range from 0 to 100, with higher scores indicating worse itch-related quality of life.

‡‡ Scores on the 5-D itch scale range from 5 to 25, with higher scores indicating worse itch-related quality of life.

### 3.1 Primary endpoint analysis

**FIGURE 2:**
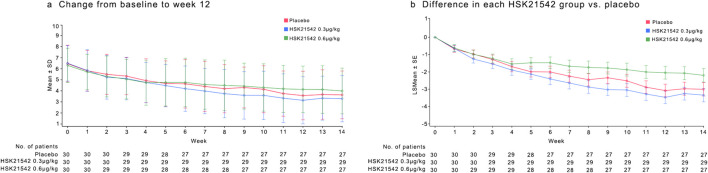
Mean of the daily Worst Itching Intensity Numerical Rating Scale WI-NRS score **(a)** and mean change in WI-NRS score **(b)** during the 12-week treatment period. **(a)** Describe the trend of the mean WI-NRS score during 12-week treatment period. **(b)** Shown is the least-squares mean change from baseline (point estimates) in the weekly mean WI-NRS score, as analyzed with the use of a mixed-effects model with repeated measures. Scores range from 0 to 10, with higher scores indicating greater intensity. The I bars indicate the standard error. Missing data were imputed with the use of multiple imputations under a missing-at-random assumption. There were 30 patients in each trial group.

The change from the baseline in the weekly mean of the daily WI-NRS scores at week 12 was −2.94, −3.40, and −2.21 for the placebo, 0.3 μg/kg, and 0.6 μg/kg HSK21542 groups, respectively. The percentage reductions from baseline were −44.26% for the placebo, −52.88% for the 0.30 μg/kg HSK21542, and -35.99% for the 0.60 μg/kg HSK21542 groups (Table 2; Figures 2a,b).

**TABLE 2:** Efficacy outcomes of HSK21542.

	Placebo (N = 30)	HSK21542 0.3 μg/kg (N = 30)	HSK21542 0.6 μg/kg (N = 30)
Weekly WI-NRS score, mean (SD)			
Baseline, mean (SD)	6.5 (1.7)	6.5 (1.6)	6.3 (1.6)
Week 12, mean (SD)	3.6 (2.2)	3.1 (2.1)	4.1 (2.1)
Change from baseline, mean (SD)	−2.9 (2.3)	−3.4 (1.9)	−2.2 (1.7)
The percentage change relative to the baseline, % (SD)	−44.3 (32.1)	−52.9 (27.9)	−36.0 (26.0)
LS, mean (SE)	−3.1 (0.4)	−3.5 (0.4)	−2.1 (0.4)
95% CI of LS mean	−3.8, −2.3	−4.2, −2.7	−2.8, −1.3
LS mean difference vs Placebo	—	−0.4	1.0
95% CI	—	−1.4, 0.6	0, 2.1
Week 13*, mean (SD)	3.7 (2.2)	3.3 (2.0)	4.1 (2.2)
Week 14*, mean (SD)	3.6 (2.2)	3.3 (2.1)	4.0 (2.1)
≥3-point improvement in the NRS score at week 12, No. (%)	12 (44.4)	18 (62.1)	10 (37.0)
≥4-point improvement in the NRS score at week 12, No. (%)	9 (33.3)	11 (37.9)	3 (11.1)
Skindex-16 scale total score			
Baseline, mean (SD)	41.6 (23.3)	44.4 (26.5)	51.6 (19.2)
Week 12, mean (SD)	23.5 (21.7)	23.4 (23.1)	28.2 (22.8)
Change from baseline, mean (SD)	−19.2 (21.4)	−22.3 (30.1)	−21.3 (18.6)
The percentage change relative to the baseline, % (SD)	−46.1 (46.8)	−17.0 (150.8)	−41.7 (38.2)
LS, mean (SE)	−21.1 (3.9)	−22.5 (3.8)	−19.8 (4.0)
95% CI of LS mean	−28.9, −13.4	−30.1, −14.9	−27.7, −11.9
LS mean difference vs Placebo	—	−1.3	1.3
95% CI	—	−12.2, 9.5	−9.8, 12.4
5-D Itch scale total score			
Baseline, mean (SD)	16.1 (3.8)	15.1 (3.8)	15.7 (3.7)
Week 12, mean (SD)	11.3 (4.7)	9.8 (4.0)	11.8 (3.7)
Change from baseline, mean (SD)	−5.0 (4.8)	−5.5 (3.9)	−3.7 (4.8)
The percentage change relative to the baseline, % (SD)	−30.0 (23.9)	−34.5 (21.5)	−20.8 (26.3)
LS, mean (SE)	−4.7 (0.7)	−5.7 (0.7)	−3.9 (0.8)
95% CI of LS mean	−6.2, −3.3	−7.2, −4.2	−5.4, −2.3
LS mean difference vs Placebo	—	−0.9	
95% CI	—	−3.0, 1.1	−1.2, 3.0

* Measurement results after discontinuation of medication, not included in primary endpoint analysis. Efficacy analyses were performed in the intention-to-treat population. Improvement was indicated by a decrease in score in all scale assessments. The primary and secondary outcomes were evaluated with the use of a prespecified hierarchical statistical testing procedure; the P value for each outcome was considered to be inferential if the preceding outcome in the sequential testing procedure was significant at a two-sided 0.05 significance level. LS, Means are adjusted means that account for baseline differences; LS, mean differences represent the adjusted differences between treatment groups, providing a more accurate reflection of the treatment effects by eliminating potential biases from baseline variations. The 95% CI, represents an estimate of the difference range of the LS, Means. 95%CI: 95% Confidence Interval, LS, mean: Least Squares Mean, SD: standard deviation, SE: standard error.

Analysis using the MMRM revealed the LS mean changes from baseline at week 12 to be −3.47 for the 0.30 μg/kg HSK21542 group and −2.05 for the 0.60 μg/kg HSK21542 group, compared with an LS mean change of −3.08 for the placebo group. The difference in LS mean changes between the 0.30 μg/kg HSK21542 and the placebo groups was −0.39 (95% CI: 1.42, 0.64), and between the 0.60 μg/kg HSK21542 and the placebo groups, it was 1.03 (95% CI: 0.01, 2.07). Although this difference was not statistically significant (*p* = 0.450), the numerical abatement in the 0.30 μg/kg HSK21542 group was greater than that in the placebo group. The results of sensitivity analyses for the primary endpoint demonstrated the consistency of the findings across different analytical approaches (Supplementary Table S1).

### 3.2 Secondary endpoints analysis

**FIGURE 3:**
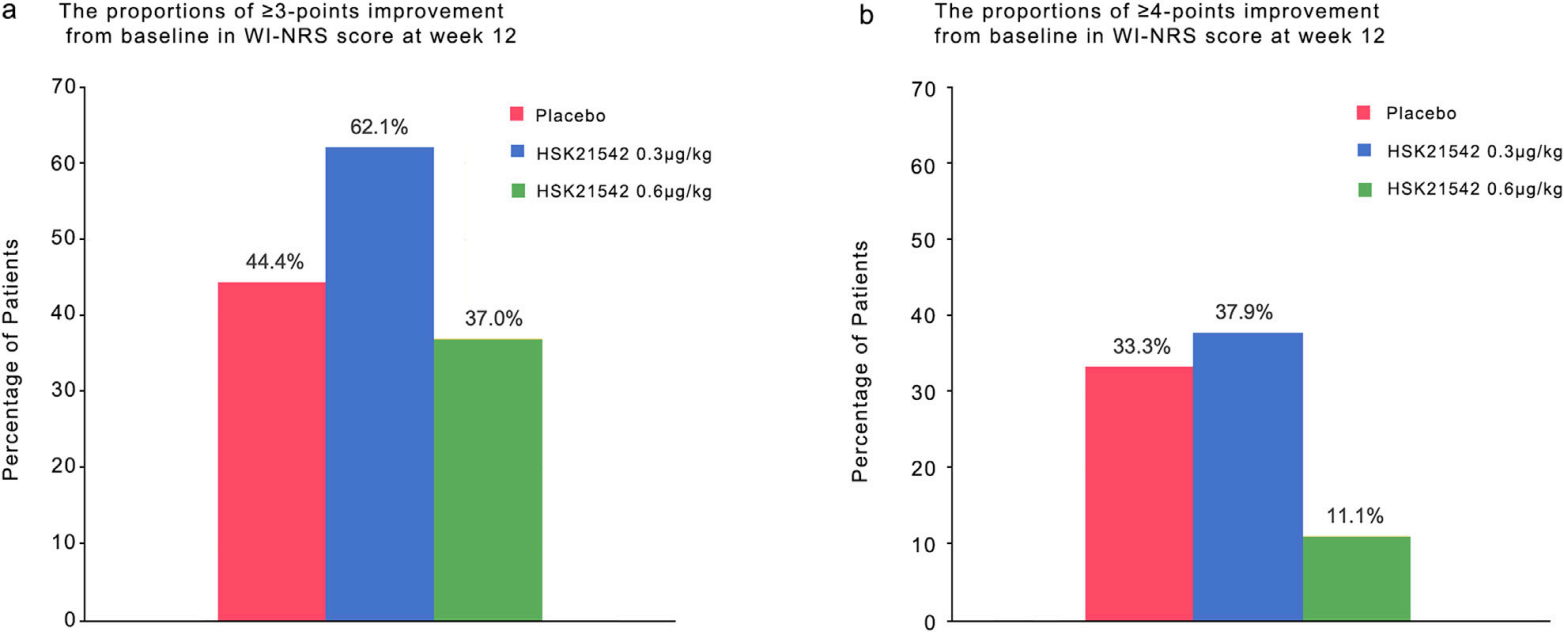
The proportions of ≥3-point and ≥4-point improvement from baseline in WI-NRS score at week 12.Panel a and b show the proportions of patients who had an improvement (decrease) of at least 3 points and of at least four points from baseline at week 12 in the weekly mean score on the daily Worst Itching Intensity Numerical Rating Scale (WI-NRS). Scores range from 0 to 10, with higher scores indicating greater intensity. A decrease of 3 points represents a clinically meaningful improvement in patients undergoing hemodialysis with moderate-to-severe pruritus; a change of four points has been estimated as the minimal clinically important difference in patients with psoriasis. There were 30 patients in each trial group.

At week 12, the fraction of participants who attained a ≥3 point reduction from baseline in WI-NRS score was 44.4% in the placebo, 62.1% in 0.30 μg/kg, and 37.0% in 0.60 μg/kg groups. A ≥ four-point reduction was observed in 33.3%, 37.9%, and 11.1% of participants, respectively (Figure 3).

**FIGURE 4:**
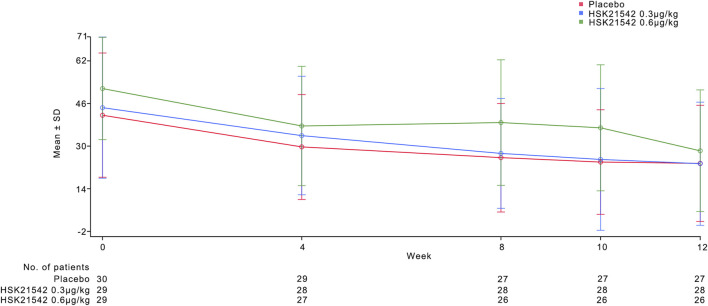
Mean of Skindex-16 score during the 12-week treatment period. Figure 4 describes the trend of the mean Skindex-16 score during 12-week treatment period.

QoL assessments using the Skindex-16 scale showed a decrease in scores with the 0.30 μg/kg group exhibiting greater improvements than the placebo group. Similar trends were observed in the 5-D Itch Scale scores (Table 2; Figure 4).

### 3.3 Exploratory analysis

The weekly mean of daily WI-NRS scores showed a consistent decrease from baseline across all treatment groups from week one to week 11. (Figures 2a,b). Post-treatment assessments of WI-NRS scores during the first 2 weeks following discontinuation of the study drug showed a reduction from baseline across all groups, maintaining a stable trend from week 12. The 0.30 μg/kg group maintained a higher reduction than the placebo group, consistent with the treatment period trend when analyzed using MMRM (Table 2). Additionally, calcium did not differ between the groups. The treatment group exhibited higher phosphate and PTH at the end of treatment (week 12) without significance, along with a milder degree of pruritus (Supplementary Figures S1-S3).

### 3.4 Safety

During the 12-week treatment period, treatment-emergent adverse events (TEAEs) were reported in 73.3% of the placebo group, 76.7% of the 0.30 μg/kg HSK21542 group, and 90.0% of the 0.60 μg/kg HSK21542 group. The most frequently reported TEAEs included dizziness, hyperkalemia, and hypotension. TEAEs led to treatment discontinuation in 13.3% of the placebo group, 3.3% of the 0.30 μg/kg group, and 13.3% of the 0.60 μg/kg group. The SAEs was observed in 12 patients, and none was considered related to the study drug. No deaths was reported throughout the study (Table 3).

**TABLE 3:** Adverse events summar**y** *.

	Placebo (N = 30)	HSK21542 0.3 μg/kg (N = 30)	HSK21542 0.6 μg/kg (N = 30)
12-week double-blinded intervention period			
TEAEs, No. (%)	22 (73.3)	23 (76.7)	27 (90.0)
Grade 1	6 (20.0)	7 (23.3)	7 (23.3)
Grade 2	7 (23.3)	12 (40.0)	12 (40.0)
Grade 3	7 (23.3)	4 (13.3)	6 (20.0)
Grade 4	2 (6.7)	0	2 (6.7)
Grade 5	0	0	0
CTCAE Grade ≥3 TEAE, No. (%)	9 (30.0)	4 (13.3)	8 (26.7)
TEAE leading to discontinuation	4 (13.3)	1 (3.3)	4 (13.3)
SAE, No. (%)	4 (13.3)	3 (10.0)	5 (16.7)
TRAE, No. (%)	5 (16.7)	5 (16.7)	5 (16.7)
Most frequently reported adverse events (≥5% in any group)^‡^			
Hyperkalemia	8 (26.7)	7 (23.3)	7 (23.3)
Hyperlipidemia	2 (6.7)	1 (3.3)	5 (16.7)
Hypocalcemia	2 (6.7)	1 (3.3)	0
Hypotension	0	2 (6.7)	1 (3.3)
Renal Anemia	6 (20.0)	7 (23.3)	5 (16.7)
Anemia	4 (13.3)	1 (3.3)	1 (3.3)
Dizziness	1 (3.3)	2 (6.7)	4 (13.3)
Muscle Spasms	2 (6.7)	1 (3.3)	2 (6.7)
Vomiting	2 (6.7)	0	2 (6.7)
Chest Discomfort	3 (10.0)	0	1 (3.3%)
2-Week discontinuation period			
TEAE, No. (%)	4 (13.3)	4 (13.3)	6 (20.0)
Most frequently reported adverse events (≥5% in any group)			
Hyperkalemia	1 (3.3)	2 (6.7)	2 (6.7)

TEAEs, Treatment-Emergent Adverse Events; TRAE, treatment related adverse events; SAE, serious adverse events

*Listed are adverse events occurring in the double-blind safety population (defined as all the patients who had undergone randomization and received at least one dose of placebo or HSK21542) between randomization and the end of the 12-week double-blind intervention period, and adverse events occurring in the 2-week discontinuation period safety population (defined as patients who had at least one visit in the discontinuation period). Preferred terms in the Medical Dictionary for Regulatory Activities, version 20.1, were used for the documentation of adverse events.

‡The most frequent adverse events during the 12-week double-blind treatment period were defined as those with an incidence of more than 5% in either trial group.

During the 2-week discontinuation observation period, hyperkalemia occurred in two cases in both the 0.30 μg/kg and 0.60 μg/kg groups, there was one case in the placebo group (Table 3).

In clinical laboratory tests, electrocardiogram, physical examinations, and vital signs are included, it was observed that there were no differences of a significant magnitude across all dosage groups and the placebo group. Notably, no withdrawal symptoms were detected post-treatment in any group. The ShOWS scores revealed an overall downward trend for all groups within 2 weeks subsequent to the discontinuation of the study medication. Compared with the placebo group, the 0.30 μg/kg HSK21542 group showed similar values at each time point, with means ranging from −1 to one point, and the overall trends were consistent. The decline in ShOWS scores for the placebo group began around 6 days post-treatment, with a slower trend compared with the 0.30 μg/kg HSK21542 group (Supplementary Figure S4). Regarding the OOWS, scores were relatively stable or showed a slight decrease from baseline across all groups, with no substantial difference noticed between the treatment and placebo groups at any visit, suggesting that physical withdrawal symptoms were comparable across all groups (Supplementary Figure S5).

### 3.5 Pharmacokinetics

Following intravenous administration of HSK21542 at various doses, the plasma concentration of HSK21542 increased with the dose. No accumulation was observed following continuous intravenous administration of HSK21542 (Supplementary Table S2; Supplementary Figure S6).

## 4 Discussion

CKD-aP, a condition that poses a potential threat of distress, exerts its influence on a considerable number of patients afflicted with CKD and ESRD, as well as those undergoing dialysis. The severity of CKD-aP manifests itself in a wide range, from experiencing sporadic bouts of discomfort to enduring unremitting restlessness throughout both the day and night. It was through the epidemiological study conducted by the Dialysis Outcomes and Practice Patterns Study (DOPPS) that the deterioration of Quality of Life (QoL) and the increased mortality associated with CKD-aP were verified (Pisoni et al., 2006; Sukul et al., 2021). Therefore, developing effective treatments for CKD-aP is crucial.

Two novel KOR agonists, nalfurafine hydrochloride and difelikefalin, have been approved for clinical use in managing CKD-P. Nalfurafine hydrochloride is a selective KOR agonist without significant activity on MOR^16.^ Several large-scale placebo-controlled studies were performed to examine the efficacy and safety of oral nalfurafine hydrochloride in treating intractable pruritus in patients undergoing hemodialysis (Kumagai et al., 2010; Zhang et al., 2023). The results showed that nalfurafine hydrochloride significantly reduced pruritus compared with the placebo group. The incidence of adverse drug reactions (ADRs) was notably higher in the treatment group. The most common ADR was insomnia, which was attributed to the activation of KOR in the central nervous system (CNS)^18^. In contrast, difelikefalin is a peripherally selective KOR agonist. Due to its low membrane permeability and curtailed transfer to CNS, it is projected to show a more favorable safety profile and improved tolerability (Narita et al., 2022). A meta-analysis evaluating the efficacy and safety of difelikefalin versus placebo in managing CKD-aP demonstrated that difelikefalin significantly reduced the weekly mean WI-NRS score, 5-D itch scale total score, and Skindex-10 total score. However, it was still associated with a higher incidence of adverse events (Saeed et al., 2024).

HSK21542, similar to difelikefalin, acts as a peripherally selective KOR agonist. This Phase II study demonstrated that continuous administration of HSK21542 over 12 weeks in patients undergoing maintenance hemodialysis could effectively alleviate CKD-aP. Notably, the 0.30 μg/kg dose significantly reduced pruritus and improved QoL scores compared to the placebo group, with sustained therapeutic effects observed post-treatment cessation.

When comparing the findings from this Phase II trial to the KALM-1 (Fishbane et al., 2020a) and KALM-2 (Wooldridge et al., 2020; Topf et al., 2022) studies, it was observed that a higher proportion of patients in the 0.30 μg/kg HSK21542 group achieved a reduction of ≥3 points in WI-NRS scores at week 12 (62.1% in HSK21542 study vs 49.1% in KALM-1, 53.4% in KALM-2). In addition, a higher proportion of patients in the placebo group experienced a reduction (44.4% in the HSK21542 study vs 27.9% in KALM-1, 42.6% in KALM-2). The proportion of patients with a reduction of ≥4 points from baseline was similar between the three studies (37.9% in HSK21542_203% vs 40.5% in KALM-1, 37.3% in KALM-2), with similar trends in the placebo group (33.3% in HSK21542 study vs 21.2% in KALM-1, 26.4% in KALM-2). The reduction in the 5-D scale total score at week 12 was also comparable between the studies for the 0.30 μg/kg HSK21542 group, with a decrease of −5.5 in HSK21542, -5.0 in KALM-1, and -4.9 in KALM-2. The treatment effect observed in the 0.6 μg/kg group was less pronounced than that in the 0.3 μg/kg group. In phase I trial, the exposure was found to be linear increase within the dose range of 0.2–3.375 μg/kg (15-min dosing duration) for HSK21542 (Shao et al., 2024). The phase II trial (stage 1) also observed a trend of increasing exposure with the dose escalation. In this trial, although various efficacy endpoints in the 0.6 μg/kg dose group showed improvement from baseline, no significant dose-response relationship was observed compared to the 0.3 μg/kg dose group. This phenomenon mirrors the findings from the phase II study of difelikefalin, where the 0.5 μg/kg dose group exhibited better efficacy than the 1 μg/kg dose group. Fresenius and Therapeutics (2021) we carefully weighed the risk-benefit profile and ultimately selected the 0.3 μg/kg dose group for further progression in phase III studies.

Multiple studies have focused on the relationship between calcium, phosphorus, parathormone (PTH), and pruritus. Hypercalcemia is associated with pruritus in most studies (Agarwal et al., 2021b; Elhag et al., 2022; Xie et al., 2022), while some evidence show opposite conclusion, possibly influenced by albumin (Hasan et al., 2020; Engler et al., 2024). Hyperphosphatemia is associated with pruritus (Agarwal et al., 2021b; Elhag et al., 2022), but some studies suggest no significant correlation (Hasan et al., 2020; Engler et al., 2024; Fishbane et al., 2024). PTH is positively correlated with pruritus (Agarwal et al., 2021b; Elhag et al., 2022; Engler et al., 2024). However, one study indicate that neither PTH, nor calcium and phosphate are associated with pruritus (Hashimoto and Okuno, 2025). Despite ongoing controversy, monitoring calcium, phosphate, and PTH has been incorporated into pruritus management strategies in CKD (Ting and Proulx, 2021; Rigatto et al., 2024). In our study, although treatment group showed relatively higher phosphate and PTH, the degree of pruritus was milder, confirming that HSK21542 may has a powerful efficacy for pruritus.

The safety profile of HSK21542 was favorable, with no significant dose-related AEs or SAEs attributed to the study drug. Notably, no withdrawal symptoms were reported following discontinuation of the drug. The second stage trial exhibited that no gastrointestinal discomfort was observed in the 0.3 μg/kg group, and no patients in treatment groups fell due to dizziness. Furthermore, the trial reinforced first stage safety results, with no new safety signals or withdrawal symptoms observed post-treatment cessation. emphasizing the drug’s well-tolerated nature.

The limitations of this study are primarily due to the lower baseline itch severity of enrolled patients and a relatively large placebo effect. Since pruritus is a subjective symptom influenced by multiple psychological factors, our results may have been significantly impacted by the placebo effect, which is a challenge commonly encountered in other studies (Ferreira et al., 2024). The intensity of itch is often correlated with the patient’s mental state and other factors, such as stress, depression, and anxiety, (Violin et al., 2014; Satti et al., 2019), which could also enhance the placebo effect (Losappio et al., 2018). Our study did not assess the mental state or related variables of patients, potentially leading to an underestimation of the efficacy of the study drug. Additionally, the relatively short 12-week treatment duration may not be sufficient to fully verify the long-term safety and effectiveness of HSK21542, suggesting that longer-term studies are necessary to provide further evidence. The small sample size also limits the generalizability of our findings. Future studies should consider larger sample sizes, longer observation periods, and a broader range of patient populations to better establish the therapeutic potential of HSK21542.

In conclusion, this Phase II clinical trial demonstrates that HSK21542 injection at a dose of 0.3 μg/kg significantly reduces pruritus and improves quality of life in hemodialysis patients, with sustained therapeutic effects observed even after treatment cessation. The safety profile of HSK21542 was favorable, with no treatment-related serious adverse events (SAEs) or deaths reported throughout the study. The 0.3 μg/kg dose exhibited superior safety and tolerability compared to the higher dose, with a lower incidence of treatment discontinuation due to adverse events. These findings suggest that HSK21542 has the potential to be an effective and well-tolerated treatment option for managing chronic kidney disease-associated pruritus (CKD-aP) in hemodialysis patients. Based on these results, further evaluation of the 0.3 μg/kg dose in Phase III clinical trials is warranted to better ascertain the long-term benefits and safety of HSK21542.

## Data Availability

The original contributions presented in the study are included in the article/Supplementary Material, further inquiries can be directed to the corresponding authors.
