# Supplementary material for: Efficacy and safety of HSK21542 for pruritus management in hemodialysis patients: a multicenter, randomized, double-blind, placebo-controlled trial
Source: Front Pharmacol. 2025 Jun 24;16:1583515. doi: 10.3389/fphar.2025.1583515 (PMC12235262; doi:10.3389/fphar.2025.1583515)
Supplement: Supplementary file 1 [file Table1.docx]

**Supplemental Table 1. Sensitivity Analyses in the Primary Endpoint**

| Method of analysis | Statistical Measure | Placebo (N=30) | HSK21542 0.3 μg/kg (N=30) | HSK21542 0.6 μg/kg (N=30) |
| --- | --- | --- | --- | --- |
| Repeated Measures Mixed-Effects Model Analysis - MI | Least Squares Mean (Standard Error) | -2.9 (0.4) | -3.4 (0.4) | -2.0 (0.4) |
|  | 95% CI | (-3.7, -2.2) | (-4.1, -2.7) | (-2.8, -1.3) |
|  | P-value | 0.6 |  |  |
|  | Least Squares Mean Difference vs. Placebo | / | -0.4 | 0.9 |
|  | 95% CI | / | -1.5, 0.6 | -0.2, 2.0 |
|  | P-value | / | 0.4 | 0.1 |
| Analysis of Covariance - OC | Least Squares Mean (Standard Error) | -2.9 (0.4) | -3.4 (0.4) | -2.3 (0.4) |
|  | 95% CI | (-3.6, -2.2) | (-4.1, -2.7) | (-3.0, -1.5) |
|  | Least Squares Mean Difference vs. Placebo | / | -0.4 | 0.7 |
|  | 95% CI | / | -1.5, 0.6 | -0.4, 1.7 |
|  | P-value | / | 0.4 | 0.2 |
| Analysis of Covariance - LOCF | Least Squares Mean (Standard Error) | -2.9 (0.3) | -3.4 (0.3) | -2.0 (0.3) |
|  | 95% CI | -3.6, -2.2 | -4.1, -2.7 | -2.7, -1.3 |
|  | Least Squares Mean Difference vs. Placebo | / | -0.5 | 0.9 |
|  | 95% CI | / | -1.5, 0.5 | -0.0, 1.9 |
|  | P-value | / | 0.3 | 0.1 |
| Analysis of Covariance - WOCF | Least Squares Mean (Standard Error) | -2.7 (0.4) | -3.3 (0.4) | -2.0 (0.4) |
|  | 95% CI | (-3.4, -2.0) | (-4.0, -2.6) | (-2.7, -1.2) |
|  | Least Squares Mean Difference vs. Placebo | / | -0.6 | 0.7 |
|  | 95% CI | / | -1.6, 0.4 | -0.3, 1.7 |
|  | P-value | / | 0.2 | 0.2 |

Logistic regression model with terms for treatment group, baseline WI-NRS score, use of anti-pruritic medication, and presence of specific medical conditions.

CI=confidence interval, LS=least-squares, MAR=missing at random, WI-NRS=Worst Itching Intensity Numerical Rating Scale.
