# Supplementary material for: Efficacy and safety of HSK21542 for pruritus management in hemodialysis patients: a multicenter, randomized, double-blind, placebo-controlled trial
Source: Front Pharmacol. 2025 Jun 24;16:1583515. doi: 10.3389/fphar.2025.1583515 (PMC12235262; doi:10.3389/fphar.2025.1583515)
Supplement: Supplementary file 2 [file Table2.docx]

**Supplemental Table 2. Descriptive Statistics of Cmax after Intravenous Bolus Injection of HSK21542 at Various Doses**

|  |  | Week 1* | Week 5* | Week 9* | Week 11* |
| --- | --- | --- | --- | --- | --- |
| HSK21542 0.3 μg/kg | Patient No. | 30 | 29 | 27 | 28 |
|  | Statistics | 3.74±1.69 (45%) | 3.52±1.46 (42%) | 3.55±1.91 (54%) | 3.52±1.73 (49%) |
| HSK21542 0.6 μg/kg | Patient No. | 30 | 28 | 27 | 27 |
|  | Statistics | 7.87±3.31 (42%) | 7.38±4.02 (54%) | 8.20±6.08 (74%) | 5.79±3.80 (66%) |

* Measurements were taken immediately after the first dose of injection.
