# Supplementary material for: Efficacy and safety of HSK21542 for pruritus management in hemodialysis patients: a multicenter, randomized, double-blind, placebo-controlled trial
Source: Front Pharmacol. 2025 Jun 24;16:1583515. doi: 10.3389/fphar.2025.1583515 (PMC12235262; doi:10.3389/fphar.2025.1583515)
Supplement: Supplementary file 3 [file Image1.pdf]

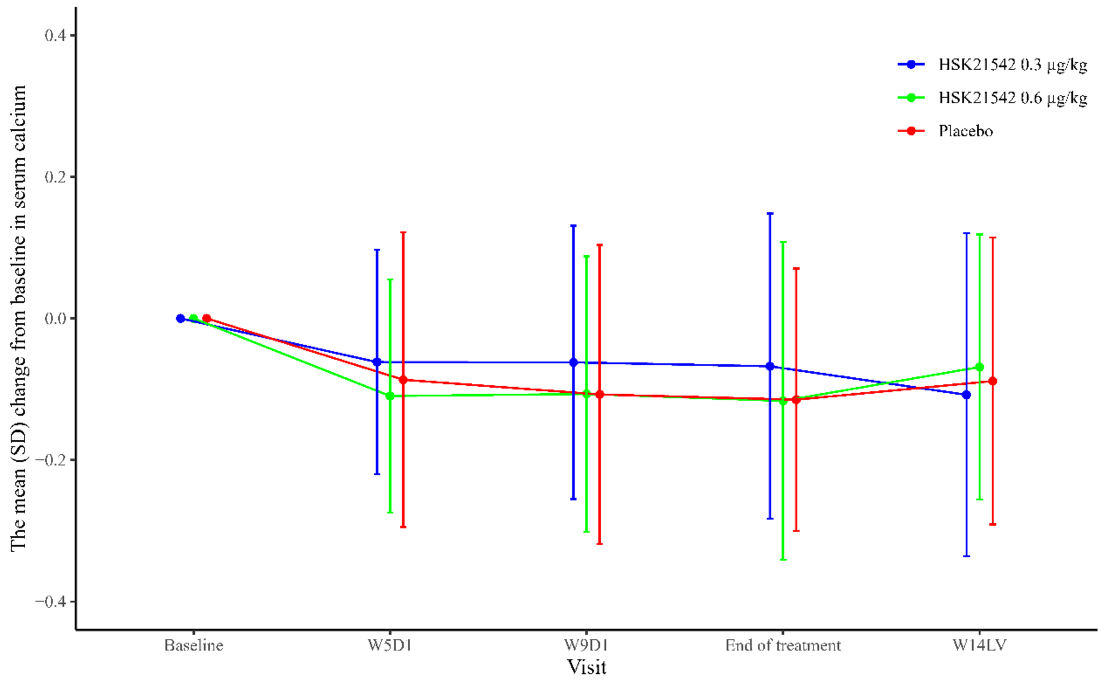

Supplementary Figure 1 Changes in Blood Calcium Levels from Baseline to Post-Treatment

A modest reduction in serum calcium levels relative to baseline without significance among groups was observed at all post-administration visits.

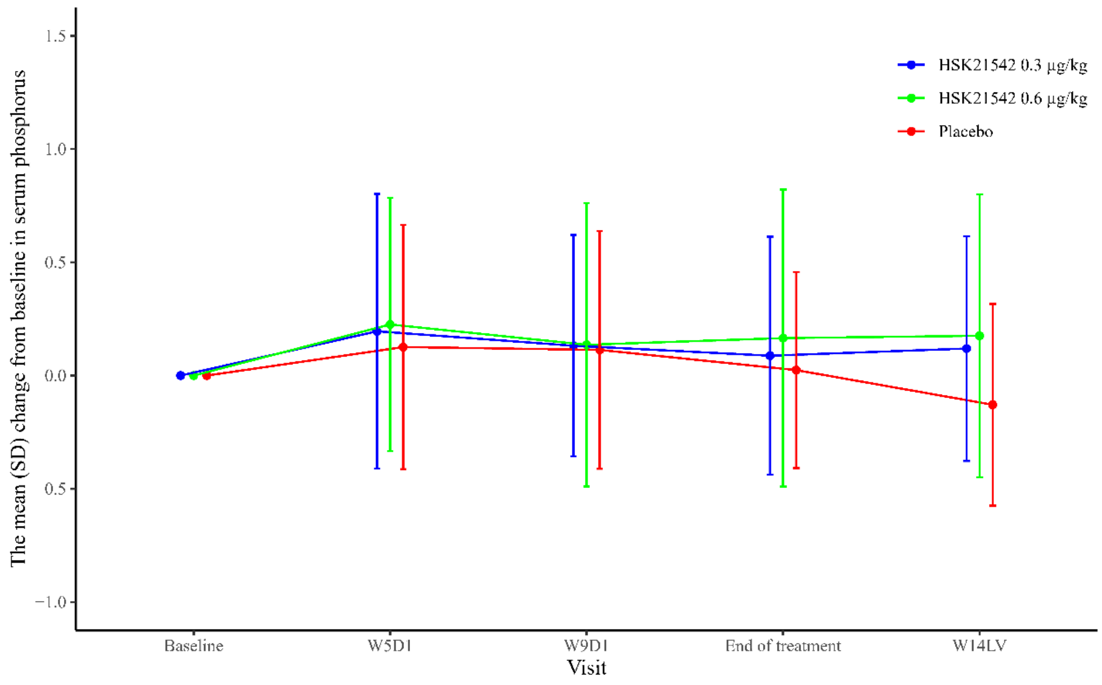

Supplementary Figure 2 Changes in Blood Phosphorus Levels from Baseline to Post-Treatment

The 0.3 µg/kg and 0.6 µg/kg groups exhibited minimal changes in serum phosphorus levels at all post-dose visits. By week 12, the placebo group showed a reduction compared to baseline, without significance among groups.

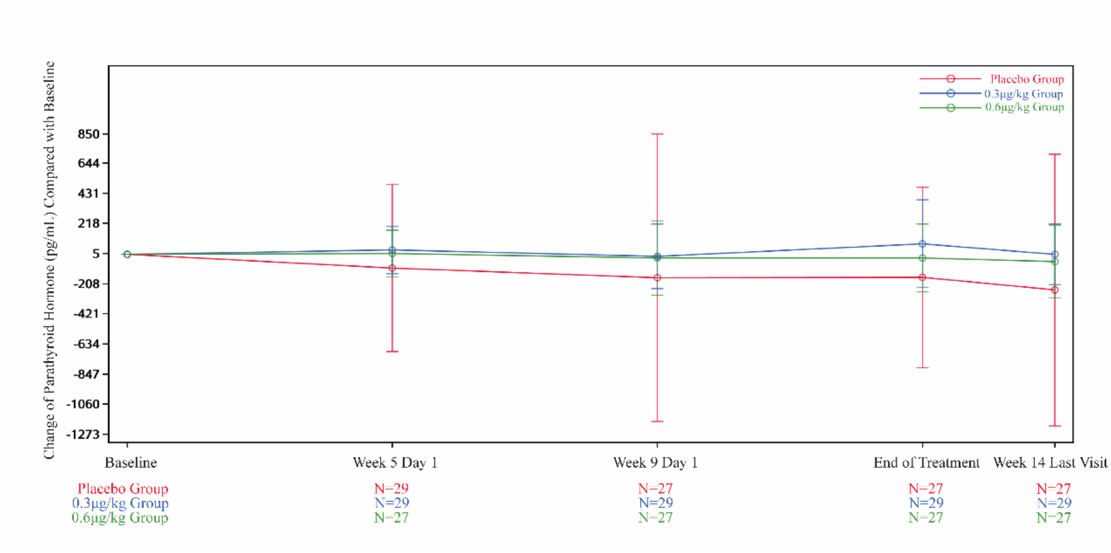

Supplementary Figure 3 Changes in PTH Levels from Baseline to Post-Treatment

PTH remained relatively stable throughout all post-administration assessments in both the 0.3 µg/kg and 0.6 µg/kg treatment arms. In contrast, the placebo group exhibited a consistent decreased trend relative to baseline. Intergroup comparisons failed to reveal any prominent differences. PTH: parathormone.

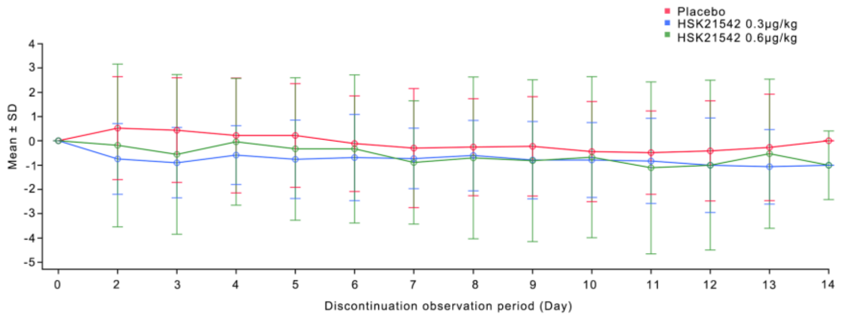

No. of patients														
Placebo	27	27	27	27	27	27	27	27	27	27	27	27	15	2
HSK21542 0.3μg/kg	29	28	29	29	29	29	29	28	29	29	29	29	15	2
HSK21542 0.6μg/kg	27	27	27	27	27	27	27	27	27	27	27	27	17	2

Note: Baseline for ShOWS and OOWS scores as the scores recorded during the final treatment visit (the first visit of Week 13)
DOP: discontinuation observation period
* On Day 13, the placebo group and the 0.3μg/kg group had 15 patients complete the follow-up, while the 0.6μg/kg group had 17 patients complete the follow-up.
** On Day 14, only two patients completed the follow-up in each group.

Supplementary Figure 4 Short Opiate Withdrawal Scale (ShOWS) Scores After Treatment Discontinuation

Mean ShOWS scores decreased over 14 days in all groups (placebo, 0.3 μg/kg, and 0.6 μg/kg HSK21542), with no significant withdrawal symptoms. The 0.3 μg/kg HSK21542 group showed consistent low scores (-1 to 1), while the placebo group had a delayed decline starting at day 6.

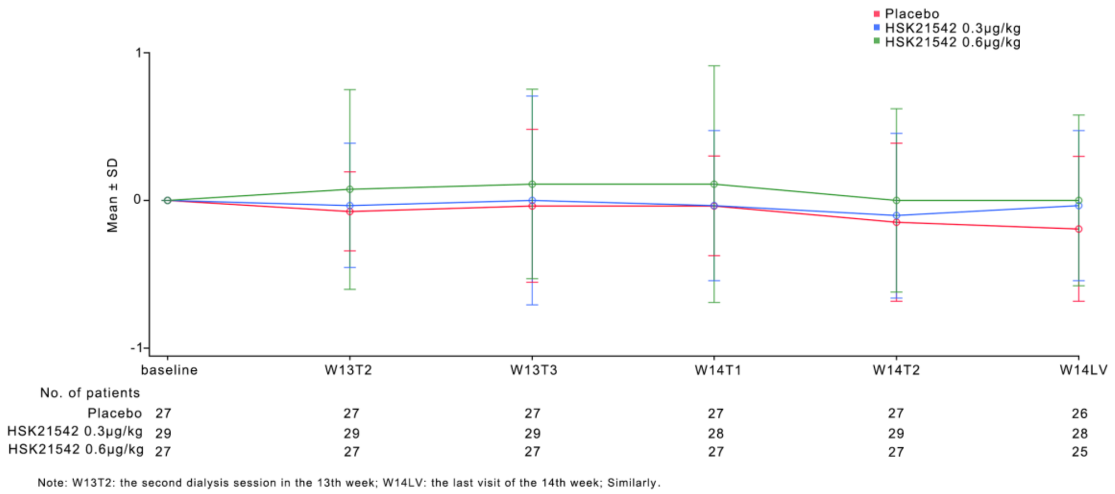

Supplementary Figure 5 Objective Opioid Withdrawal Scale (OOWS) Scores During Post-Treatment Follow-Up

OOWS scores remained stable or slightly decreased from baseline in all groups post-treatment, with no significant differences between HSK21542 doses and placebo. Results suggest no clinically meaningful physical withdrawal symptoms across groups.

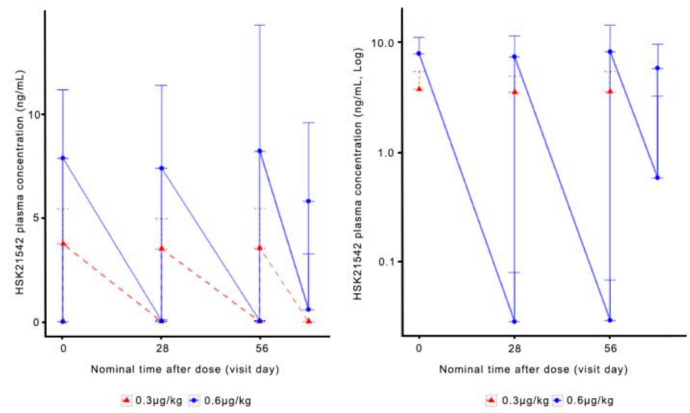

Supplementary Figure 6 Pharmacokinetics of HSK21542 After Repeated Intravenous Administration

Plasma concentrations of HSK21542 increased dose-dependently (0.3 µg/kg and 0.6 µg/kg) without accumulation over 12 weeks. Linear pharmacokinetics were observed, with stable maximum concentration (C_{max}) and area under the curve (AUC) across doses.
